# Bilateral Parsonage–Turner Syndrome in a Patient With Hemophagocytic Lymphohistiocytosis

**DOI:** 10.1155/crnm/6652600

**Published:** 2025-07-21

**Authors:** Nader Pahlevan, Delora E. Denney, Ezekiel Gonzalez-Fernandez, Oriana Sanchez, Mark Anderson

**Affiliations:** ^1^Department of Neurology, University of Mississippi Medical Center, Jackson, Mississippi, USA; ^2^Department of Neurology, Tulane Medical Center, New Orleans, Louisiana, USA

**Keywords:** brachial plexopathy, hemophagocytic lymphohistiocytosis, Parsonage–Turner

## Abstract

**Introduction:** Parsonage–Turner syndrome (PTS), also known as idiopathic brachial plexopathy, is an uncommon peripheral neuropathy, and the presentation of bilateral PTS is even rarer. Hemophagocytic lymphohistiocytosis (HLH) is a hyperinflammatory disorder that is normally considered hematologic but can involve the nervous system in up to 70% of cases.

**Case Presentation:** A 56-year-old Caucasian female with a history of SLE, rheumatoid arthritis, Sjogren's disease, and Hashimoto's thyroiditis, but no prior neurologic history, was admitted for the management of isolated thrombocytopenia, diagnosed with HLH, and then developed bilateral upper extremity pain, weakness, and numbness. A diagnosis of bilateral PTS was supported by magnetic resonance imaging (MRI) findings of mild symmetric increased enhancement in the bilateral shoulder muscles, and electromyography/nerve conduction study (EMG/NCS) revealed active denervation in the shoulder muscles bilaterally. The patient was started on methylprednisolone 1000 mg for three days, followed by a long steroid taper along with physical/occupational therapy with significant improvement of her symptoms.

**Conclusion:** It is important to maintain a high index of suspicion for PTS in patients with new-onset shoulder or upper arm pain, weakness, and sensory deficits, even if findings are bilateral. Concomitant inflammatory disorders, infection, and recent surgeries/procedures should prompt a high degree of suspicion of this disorder, and the use of relevant diagnostics, such as MRI brachial plexus and EMG/NCS, should help guide diagnosis, as this condition is very responsive to treatment.

## 1. Introduction

Parsonage–Turner syndrome (PTS), first described in *The Lancet* in 1948 by English physicians Maurice Parsonage and Aldren Turner, has roots in medical literature dating back to 1897 [1, 2]. These early descriptions of PTS occurred even before the characterization of Guillain–Barré syndrome [2]. In their seminal paper, Parsonage and Turner reported on 136 World War II soldiers who developed brachial plexus inflammation, with the majority (71%) having a history of recent infection, surgical procedures, or trauma [2].

PTS, also known as idiopathic brachial plexopathy, is an uncommon peripheral neuropathy that typically presents with sudden onset unilateral shoulder pain, followed by weakness, numbness, and dysesthesias. Pain is usually reported to be more bothersome at night, often interrupting sleep. PTS can also include specific nerve involvement from the phrenic nerve causing dyspnea and the long thoracic nerve and/or the spinal accessory nerve causing winged scapula. Interestingly, PTS is the leading cause of winged scapula [2].

The estimated incidence of PTS was previously thought to be 2-3 in 100,00, but a 2015 study in the Netherlands reported an incidence of 100 in 100,000, suggesting it could be more common than previously observed. Bilateral presentations of PTS are even rarer, estimated to comprise only one-third of all PTS cases [2] and can therefore contribute to diagnostic complexity.

Viral illnesses, specifically COVID-19, immunizations, surgeries, minor procedures such as lumbar punctures, and anesthesia are all common risk factors for the development of PTS [2]. PTS is thought to be triggered by inflammatory/immune-mediated mechanisms, but the exact pathogenesis is still unclear. No literature to our knowledge has shown that infectious products infiltrate the nerves, but rather it is proposed that an antigenic trigger causes an inflammatory response in the nervous tissue [1, 2]. Another theory is that PTS is caused by axonal degeneration secondary to microvasculature inflammation which has been reported in autoimmune diseases such as systemic lupus erythematosus (SLE), temporal arteritis, and polyarteritis nodosa [1, 2]. However, to date, there are only a handful of case reports of PTS in the setting of autoimmune diseases [1, 2].

Functional recovery after PTS has been reported to be favorable, as most studies report that patients generally return to baseline at 3 years. However, it is important to note that the studies do not typically reassess if muscle reinnervation/recruitment was observed with repeat electromyography/nerve conduction study (EMG/NCS), so improvement in functionality could also be attributed to compensatory changes [1].

## 2. Case Report/Case Presentation

We report a 56 years-old Caucasian female with a history of SLE, rheumatoid arthritis, Sjogren's disease, and Hashimoto thyroiditis, but no prior neurologic history, who was admitted for the management of isolated thrombocytopenia and acute renal failure and subsequently developed bilateral upper extremity shoulder pain, weakness, and numbness after a complicated hospital course. The patient was also recently diagnosed with hemophagocytic lymphohistiocytosis (HLH) for which she was started on etoposide, cyclosporine, and dexamethasone. The patient was admitted to the ICU for over 3 weeks and intubated. After extubation, she noticed generalized weakness in all four extremities. She retained reflexes in her bilateral Achilles tendons and right patellar. A few days later, the weakness in her legs improved, but she remained unable to move both arms. Symptoms were suspected to be attributed to myopathy secondary to steroid treatment, so steroids were discontinued. However, the patient's bilateral shoulder pain and weakness did not improve. Neurology was consulted [3].

Other more common disorders with similar presentations were reasonably excluded. Chronic inflammatory demyelinating polyneuropathy (CIDP) is a neurologic disorder characterized by progressive proximal and distal body weakness and generalized areflexia [4]. The patient's body weakness had a sudden onset, was centered in the upper extremities, and she had both Achilles and the right patellar reflexes present. Guillain–Barré is a neurologic disorder that can occur following infection or immunization but is marked by ascending paralysis and areflexia [5]. The paralysis presented uniformly, followed by being concentrated in the upper extremities; Guillain–Barré is also reasonably excluded by the absence of areflexia.

### 2.1. Labs

ESR and CRP were slightly elevated. Complete blood count fluctuated throughout the admission with WBCs ranging from 0.1 to 63,000. TSH ranged from 0.15 to 5.60 with a free T4 of 2.25. Comprehensive metabolic panel, hepatic function panel, CSF studies, CK, vitamins B1 and B12, heavy metals, and ganglioside antibodies were obtained and were all unremarkable [3].

### 2.2. Neurological Exam

Cranial nerves II-XII were intact. The exam revealed decreased tone of bilateral upper extremities and 1/5 strength in a “man-in-a-barrel” presentation. There was sensory impairment to temperature and pinprick involving both arms. No dermatomal distribution or sensory level loss on the upper extremities was noted and vibratory sensation remained intact. Mild proximal weakness was noted on the lower extremities without sensory impairment [3].

### 2.3. Neurological Examination Summary

#### 2.3.1. Initial Neurology Evaluation

Cranial nerves II–XII were grossly intact. The patient exhibited decreased muscle tone and symmetric weakness of the upper extremities, consistent with a “man-in-the-barrel” presentation. Strength in the bilateral deltoids, biceps, triceps, grip, and dorsal interossei (DIO) were 1/5. Lower extremity strength was relatively preserved, with proximal muscle groups (iliopsoas, quadriceps, hamstrings) rated at 3/5 and distal muscle groups (tibialis anterior and gastrocnemius) at 5/5. Deep tendon reflexes were absent in the biceps, triceps, and brachioradialis bilaterally. Patellar reflex was diminished on the right and absent on the left; ankle reflexes were present but diminished (1+ bilaterally). Sensory examination revealed decreased temperature and pinprick sensation in both upper extremities without a dermatomal pattern or sensory level, while vibratory sensation remained intact. The patient reported severe pain rated 10/10 that was predominantly in the shoulders bilaterally but with some proximal arm involvement and worst at night, which likely contributed to some degree to the motor exam.

#### 2.3.2. Follow-Up Examination (1 week later)

Six days after initiating intravenous corticosteroids, the patient reported subjective improvement in strength. On examination, there was a slight increase in proximal upper extremity strength, with deltoids now being 2/5 bilaterally, while biceps, triceps, grip, and DIO remained at 1/5. Sensory exam showed improvement, with intact light touch and pinprick sensation in both arms.

#### 2.3.3. Subsequent Follow-Up (3 more weeks later)

On the last day, the patient was examined by the neurology service, and grip and distal hand motor function improved to 3/5 bilaterally. Deltoid strength remained at 2/5 bilaterally, but biceps and triceps remained at previously noted 0/5 bilaterally, suggesting differences in proximal versus distal recovery course. The patient now only experiences mild pain.

### 2.4. Neurodiagnostics

**Figure 1:**
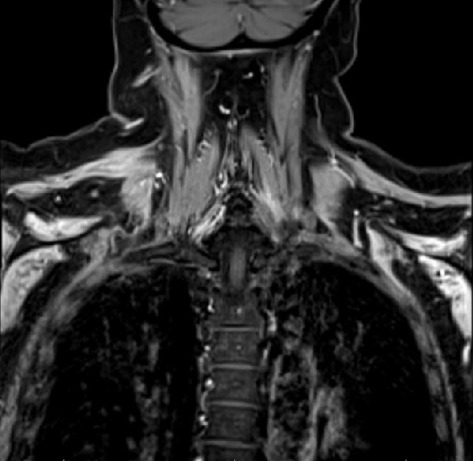
T1 MRI of the brachial plexus (limited by body habitus and movement) reveals diffuse atrophy of the shoulder musculature and homogeneous mild increased enhancement in the shoulder musculature bilaterally.

**Figure 2:**
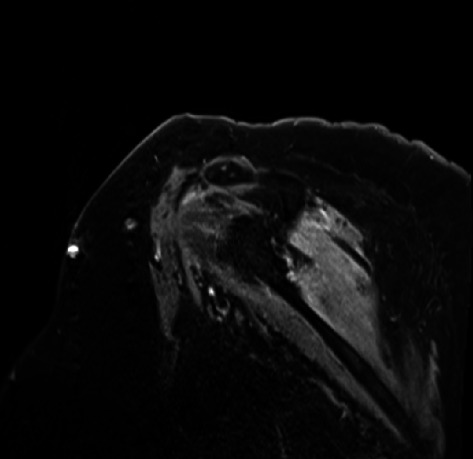
T1 MRI of the right brachial plexus reveals mild increased enhancement in the shoulder musculature.

The diagnosis of PTS is mostly by EMG but can be supported with magnetic resonance imaging (MRI). EMG/NCS testing of our patient revealed active denervation in bilateral shoulder and arm muscles, specifically denoted by fibrillation potentials and early reinnervation of motor units in the right triceps, both first dorsal interossei and left flexor carpi radialis indicative of patchy healing [1]. The right and left deltoids and right biceps' muscles all failed to demonstrate activity, implicating the right and left axillary nerve and the right musculocutaneous nerve (Table 1). Signal hyperintensities, specifically on T2 MRI, are a hallmark finding for PTS, and musculature abnormalities may also be present. However, some patients with PTS can have normal MRI findings, and thus absence of MRI findings should not preclude a diagnosis of PTS [6, 7]. Our patient had MRI imaging of the brachial plexus, revealing homogeneous mild increased enhancement in the shoulder musculature bilaterally (Figures 1 and 2).

### 2.5. Treatment

The patient was started on methylprednisolone 1000 mg for three days followed by a long steroid taper and completed physical/occupational therapy. She was also given nortriptyline at night for pain. Robust evidence for steroid treatment is lacking; anecdotally, it can shorten PTS duration. However, the main goal of steroid administration is pain control as opposed to inflammation reduction [1, 2]. Hydromorphone was given as needed for pain control.

### 2.6. Hematology

HLH is a hyperinflammatory disorder that is normally considered hematologic but can involve the nervous system in up to 70% of cases [6]. The incidence is one in 2000 for adult critical care admissions. HLH is caused by the dysregulation of natural killer (NK) cells and CD8+ cytotoxic T cells, leading to an inflammatory cascade. Diagnosis can be primary, which is normally familial and inherited, or secondary due to an acute illness trigger or autoimmune disorder [4]. The patient met the 2004 criteria for HLH, with 5/8 factors being required for diagnosis. She was febrile and her labs reflected bicytopenia, hypertriglyceridemia, low or absent NK cell activity, and a ferritin level greater than 500 [6]. Her subtype is assumed to be secondary, which commonly presents in the fifth decade of life [6].

## 3. Discussion

This is the first case report of PTS in the setting of HLH to our knowledge. It seems plausible that PTS could have been a manifestation of HLH, especially since bilateral presentations are rarer and HLH is a systemic process. As HLH is an inflammatory disease, this would give support to the theory of axonal degeneration due to an inflammatory process as a potential etiology of PTS. One week before the patient's PTS diagnosis, her WBC count was 63,000. When she received her PTS diagnosis, her ESR was 34 and her CRP was 0.8. These findings suggest inflammation, although it cannot be attributed to one specific cause. We recognize that the patient's history of SLE also confounds the etiology.

The patient also had many other risk factors that have been previously associated with PTS. She was septic with positive blood cultures in the month leading up to her PTS diagnosis. She was subsequently intubated and sedated, exposing her to anesthetics. She underwent a lumbar puncture 2 weeks prior.

## 4. Conclusion

PTS is extremely rare, and bilateral presentations are even more uncommon making diagnosing this condition particularly challenging. It is important to keep PTS on the differential diagnosis in patients with axillary, shoulder, or arm pain and subsequent weakness and/or sensory deficits. Concomitant inflammatory disorders, infection, and recent surgeries/procedures should prompt a high degree of suspicion of this disorder, and the use of relevant diagnostics, such as EMG/NCS and MRI brachial, may help guide diagnosis and treatment. Prior case reports have described neurological manifestations of HLH, which raises suspicion for HLH as a possible inciting etiology of PTS [8].

It is not possible to conclude the exact cause of bilateral PTS in this patient. However, we hope that by reporting this case and the unique comorbid conditions associated, we will help contribute to the growing body of literature on PTS and HLH and provide additional clues into the mechanistic etiology of this disorder.

## Figures and Tables

**Table 1:** Evidence of a bilateral brachial plexopathy that is severe and extensive in both arms with early reinnervation of motor units noted on EMG in the right triceps, both first dorsal interossei and left flexor carpi radialis indicative of early patchy healing.

EMG summary table										
Muscle	Spontaneous					MUAP				Recruitment
	IA	Fib	PSW	Fasc	Others	Amp	Dur.	PPP	Effort	Pattern
R. Deltoid	1+	3+	3+	None	None					No activity
R. Biceps brachii	1+	3+	4+	None	None					No activity
R. Triceps brachii	1+	4+	None	None	None	N	N	3+	N	Reduced
R. First dorsal interosseous	1+	3+	3+	None	None	N	N	3+	N	Reduced
R. Extensor indicis proprius	1+	3+	3+	None	None					
R. Vastus lateralis	N	None	None	None	None	N	N	N	N	N
L. Deltoid	1+	3+	3+	None	None					No activity
L. Biceps brachii	1+	3+	3+	None	None					
L. Triceps brachii	1+	4+	4+	None	None					
L. Flexor carpi radialis	1+	3+	3+	None	None	N	N	3+	N	Reduced
L. First dorsal interosseous	1+	3+	3+	None	None	Sl. Inc.	Sl. Inc.	3+	N	Reduced
L. Serratus anterior	N	None	None	None	None	N	N	N	N	N
R. Serratus anterior	N	None	None	None	None	N	N	N	N	Discrete

## Data Availability

The data that support the findings of this study are available from the corresponding author upon reasonable request.
